# Gremlin promotes retinal pigmentation epithelial (RPE) cell proliferation, migration and VEGF production via activating VEGFR2-Akt-mTORC2 signaling

**DOI:** 10.18632/oncotarget.13518

**Published:** 2016-11-23

**Authors:** Yuan Liu, Zhijun Chen, Haixia Cheng, Juan Chen, Jing Qian

**Affiliations:** ^1^ Department of Ophthalmology, Nanjing First Hospital, Nanjing Medical University, Nanjing, China; ^2^ Department of Ophthalmology, Children's Hospital of Nanjing Medical University, Nanjing, China

**Keywords:** retinopathy of prematurity (ROP), gremlin, retinal pigmentation epithelial (RPE), VEGF and signaling

## Abstract

Retinopathy of prematurity (ROP) is characterized by late-phase pathologic retinal vasoproliferation. Gremlin is a novel vascular endothelial growth factors (VEGF) receptor 2 (VEGFR2) agonist and promotes angiogenic response. We demonstrated that gremlin expression was significantly increased in retinas of ROP model mice, which was correlated with VEGF upregulation. In retinal pigmentation epithelial (RPE) cells, gremlin activated VEGFR2-Akt-mTORC2 (mammalian target of rapamycin complex 2) signaling, and promoted cell proliferation, migration and VEGF production. VEGFR inhibition (by SU5416) or shRNA knockdown almost abolished gremlin-mediated pleiotropic functions in RPE cells. Further, pharmacological inhibition of Akt-mTOR, or shRNA knockdown of key mTORC2 component (Rictor or Sin1) also attenuated gremlin-exerted activities in RPE cells. We conclude that gremlin promotes RPE cell proliferation, migration and VEGF production possibly via activating VEGFR2-Akt-mTORC2 signaling. Gremlin could be a novel therapeutic target of ROP or other retinal vasoproliferation diseases.

## INTRODUCTION

Retinopathy of prematurity (ROP) is one important reason of childhood blindness [1–3]. The use of supplemental oxygen in closed incubators for the preterm infants is one major cause of ROP [3]. ROP starts from an arrest of retinal vascularisation (phase 1), which is followed by later hypoxia-induced pathologic vasoproliferation (phase 2) [1–4]. It will eventually lead to a complete retinal detachment behind the lens [3]. The detailed pathological mechanisms of ROP are still debatable [3]. Further, due to the invasive nature of this disease, current ROP clinical treatments are far from satisfactory in improving prognosis [3].

Gremlin, a highly conserved protein, has a total of 184 amino acid with a cysteine rich region [5–8]. It is a member of the cysteine knot superfamily, which can present in both soluble and cell associated forms [9–12]. Gremlin is a novel family of bone morphogenetic protein (BMP) antagonist [5]. Its function could be post-translationally modified through glycosylation and/or phosphorylation [5, 9–13]. Existing evidences have shown that gremlin could affect diverse cellular functions, including growth, differentiation, and development [5, 9–13].

Recent studies have proposed a critical function of gremlin in vasoproliferation [13]. It has been shown that gremlin is a novel agonist of vascular endothelial growth factors (VEGF) receptor 2 (VEGFR2) [6, 13], the latter is the main receptor of VEGF-mediated angiogenic signals [6, 13, 14]. Gremlin binding to VEGFR2 will activate downstream signalings and lead to VEGFR2-dependent angiogenic response [13]. In the current report, we show that gremlin expression is significantly upregulated in retinas of ROP model mice. Our *in vitro* studies in retinal pigmentation epithelial (RPE) cells show that gremlin promotes cell proliferation, migration and VEGF production via activating VEGFR2 signaling.

## RESULTS

### Upregulation of gremlin and VEGF in the retinas of retinopathy of prematurity (ROP) model mice

**Figure 1:**
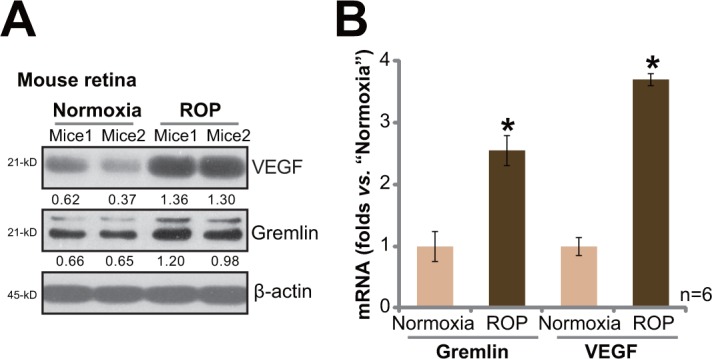
Upregulation of gremlin and VEGF in the retinas of retinopathy of prematurity (ROP) model mice Protein and mRNA expressions of gremlin and VEGF in retinas of ROP model mice (P17) and control normoxia mice (P17) were tested by Western blot assay **A.** and RT-qPCR assay **B.**, respectively. For the RT-qPCR assay, n=6. Gremlin and VEGF protein expression (vs. β-actin) was quantified (A). Experiments in this figure were repeated three times, and each time similar results were obtained. Bars ± error bars stand for mean ± SD (Same for all figures). * *p* < 0.05 *vs.* “Normoxia” group (B).

First, we tested expressions of gremlin in ROP model mice. As described, C57BL/6J mice at P7 were first exposed to 75% oxygen for a total of 5 days (P7-P12). Afterwards, mice were kept in normoxia for additional 5 days. At P17, the retinas were isolated and lysed. Western blot assay results showed that, compared to the control normoxia mice, and expressions of gremlin and VEGF in retinas of ROP mice were significantly upregulated (Figure 1A). Quantification data demonstrated that gremlin protein expression nearly doubled after ROP (Figure 1A). Further, real-time quantitative PCR (“RT-qPCR”) assay results showed that gremlin and VEGF mRNA expressions were also increased in ROP mice retinas (Figure 1B), as compared to that in control mice. These results show that gremlin expression is increased in ROP mice, and its level is correlated with VEGF upregulation.

### Gremlin promotes APRE-19 cell proliferation, migration and VEGF production

**Figure 2:**
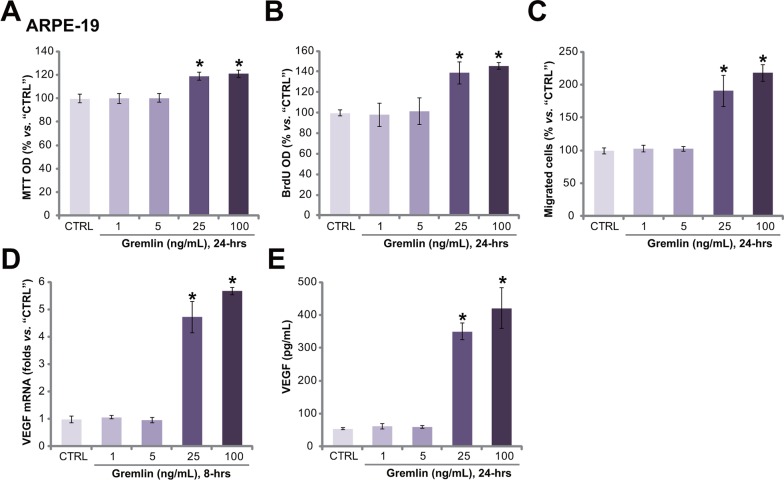
Gremlin promotes APRE-19 cell proliferation, migration and VEGF production APRE-19 cells were treated with applied concentrations of gremlin (1-100 ng/mL) for indicated time, cell proliferation was tested by MTT assay **A.** or BrdU ELISA assay **B.** Cell migration was evaluated by the “Transwell” assay **C.** VEGF mRNA expression and protein secretion were examined by RT-qPCR assay **D.** and ELISA assay **E.**, respectively. For each assay, n=5. Experiments in this figure were repeated three times, and each time similar results were obtained. “CTRL” stands for untreated control group (Same for all following figures). * *p* < 0.05 *vs.* “CTRL” group.

To test the potential effect of gremlin *in vitro*, we treated APRE-19 cells with gremlin. Methylthiazol tetrazolium (MTT) assay and 5-bromo-2'-deoxyuridine (BrdU) incorporation ELISA assay were performed to test cell proliferation. Results from these assays showed that gremlin at 25 and 100 ng/mL promoted ARPE-19 cell proliferation (Figure 2A and 2B). The MTT OD and BrdU intensity in ARPE-19 cells were increased following gremlin treatment (Figure 2A and 2B). Notably, lower concentrations of gremlin (1 and 5 ng/mL) had no significant effect on ARPE-19 cell proliferation (Figure 2A and 2B). Next, the potential effect of gremlin on RPE cell migration was also tested by the “Transwell” assay [15]. The quantified results in Figure 2C showed that gremlin dose-dependently increased the number of migrated ARPE-19 cells, confirming its pro-migration activity. Further studies showed that gremlin (25 and 100 ng/mL) treatment in APR-19 cells also promoted VEGF mRNA expression (Figure 2D) and protein secretion (Figure 2E). Lower concentrations of gremlin (1 and 5 ng/mL) again were in-effective on VEGF (Figure 2D and 2E). Together, these results demonstrate that gremlin dose-dependently promotes APRE-19 cell proliferation, migration and VEGF production.

### VEGFR2 activation is required for gremlin-exerted pleiotropic functions in ARPE-19 cells

**Figure 3:**
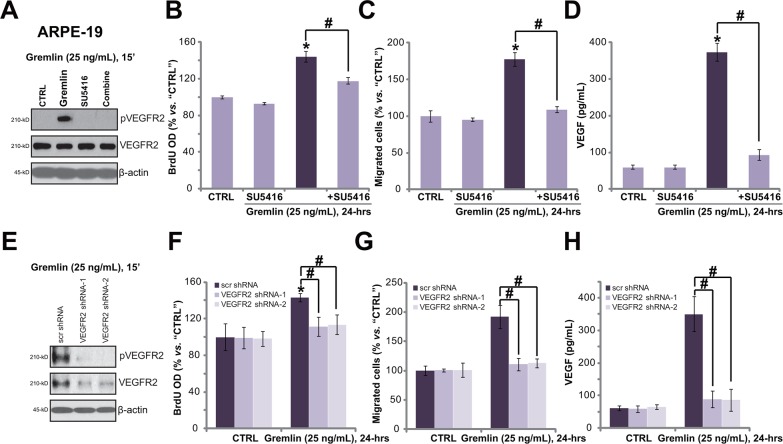
VEGFR2 activation is required for gremlin-exerted pleiotropic functions in ARPE-19 cells APRE-19 cells were treated with gremlin (25 ng/mL) or plus SU5416 (500 nM) for indicated time, VEGFR2 expression/phosphorylation were tested by Western blot assay **A.**; Cell proliferation (BrdU ELISA assay, **B.**), migration (“Transwell” assay, **C.**) and VEGF production (ELISA assay, **D.**) were also tested. The stable APRE-19 cells expressing the scramble control non-sense shRNA (“scr shRNA”) or different VEGFR2 shRNA (“-1/−2”) were treated with gremlin (25 ng/mL) for applied time, VEGFR2 expression/phosphorylation **E.**, cell proliferation **F.**, migration **G.** and VEGF production **H.** were tested. Experiments in this figure were repeated three times, and each time similar results were obtained. *p < 0.05 *vs.* “CTRL” group. ^#^ *p* < 0.05.

Recent evidences have confirmed that gremlin is a novel agonist of VEGFR2 [6, 13]. We next wanted to know the possible involvement of VEGFR2 in gremlin-exerted functions in RPE cells. Western blot results in Figure 3A showed that gremlin (25 ng/mL) treatment in APRE-19 cells induced profound VEGFR2 phosphorylation. The expression of regular VEGFR2 was not affected (Figure 3A). Remarkably, SU5416, a small molecule inhibitor of VEGFR [16] (Figure 3A), almost blocked gremlin-induced VEGFR2 phosphorylation (Figure 3A), APRE-19 cell proliferation (BrdU ELISA assay, Figure 3B), migration (“Transwell” assay, Figure 3C) and VEGF production (ELISA assay, Figure 3D). These results imply that activation of VEGFR2 is required for gremlin-exerted functions in ARPE-19 cells.

Since SU5416 is pan and non-selective VEGFR inhibitor [16], we next applied the shRNA strategy to selectively knockdown VEGFR2. As demonstrated, the two non-overlapping VEGFR2 shRNAs (“-1/−2”) dramatically downregulated VEGFR2 expression in ARPE-19 cells (Figure 3E). Importantly, gremlin-induced VEGFR2 phosphorylation (Figure 3E), cell proliferation (Figure 3F), migration (Figure 3G) and VEGF production (Figure 3H) were almost abolished in VEGFR2-silenced cells. These results again show that VEGFR2 activation is required for gremlin-induced pleiotropic functions in ARPE-19 cells. Notably, VEGFR2 shRNAs alone showed no effect on APRE-19 cells (Figure 3E–3H).

### Gremlin activates VEGFR2, and promotes cell proliferation, migration and VEGF production in primary RPE cells

**Figure 4:**
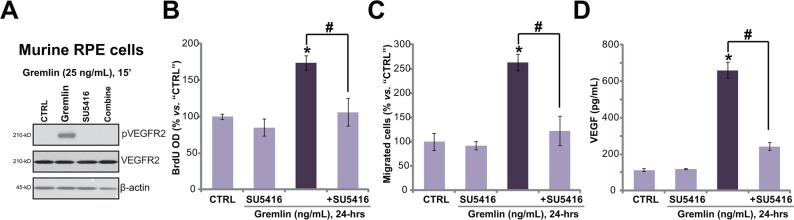
Gremlin activates VEGFR2, and promotes cell proliferation, migration and VEGF production in primary RPE cells Primary cultured murine RPE cells were treated with gremlin (25 ng/mL) or plus SU5416 (500 nM) for applied time, VEGFR2 expression/phosphorylation (Western blot assay, **A.**), cell proliferation (BrdU ELISA assay, **B.**), migration (“Transwell” assay, **C.**) and VEGF production (ELISA assay, **D.**) were tested. *p < 0.05 *vs.* “CTRL” group. ^#^ *p* < 0.05.

The potential effect of gremlin on primary murine RPE cells was also tested. As demonstrated, treatment of gremlin (25 ng/ml) provoked significant VEGFR2 phosphorylation in the primary cultured murine RPE cells (Figure 4A). Further, gremlin promoted cell proliferation (Figure 4B), migration (Figure 4C) and VEGF production (Figure 4D) in the primary cells. Notably, co-treatment with the VEGFR inhibitor SU5416 almost completely blocked gremlin-mediated pleiotropic functions in the primary RPE cells (Figure 4A–4D). These results again imply that VEGFR2 activation is indispensable for gremlin-mediated activities in the primary RPE cells.

### Akt-mTOR activation is required for gremlin-induced pleiotropic functions in RPE cells

**Figure 5:**
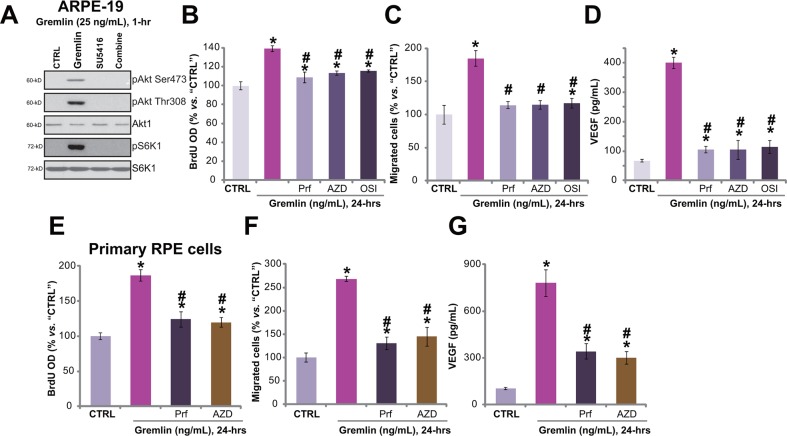
Akt-mTORC2 activation is required for gremlin-induced pleiotropic functions in RPE cells APRE-19 cells were treated with gremlin (25 ng/mL) or plus SU5416 (500 nM) for 1 hour, expressions of listed proteins were tested by Western blot assay **A.** APRE-19 cells **B-D.** or primary murine RPE cells **E-G.** were pre-treated for 30 min with the Akt specific inhibitor perifosine (“Prf”, 10 μM), mTOR kinase inhibitor AZD8055 (“AZD”, 500 nM) or OSI-027 (“OSI”, 500 nM), cells were then treated with gremlin (25 ng/mL) for 24 hours, BrdU incorporation (B, and E), migration (C and F) and VEGF production (D and G) were tested. Experiments in this figure were repeated three times, and similar results were obtained. * *p* < 0.05 *vs.* “CTRL” group. ^#^ *p* < 0.05 *vs.* gremlin only treatment.

The above results have demonstrated that gremlin activated VEGFR2 to promote RPE cell proliferation, migration and VEGF production. Next, the downstream signalings of VEGFR2 that possibly mediate gremlin's functions were analyzed. Results showed that gremlin (25 ng/mL) treatment in ARPE-19 cells induced significant phosphorylations of Akt (Ser-473 and Thr-308) and S6K1 (Thr-389) (Figure 5A), indicating significant activation of Akt-mTOR (mammalian target of rapamycin) signaling. Co-treatment with the VEGFR inhibitor SU5416 almost completely blocked Akt-mTOR activation by gremlin (Figure 5A), suggesting that Akt-mTOR lies downstream of VEGFR2. To study the role of Akt-mTOR signaling in gremlin-exerted pleiotropic functions, pharmacological strategy was first applied. The results demonstrated that the Akt-specific inhibitor perifosine [17] and the mTORC1/2 kinase inhibitor (AZD8055 [18] or OSI-027 [19]) attenuated gremlin-induced APRE-19 cell proliferation (Figure 5B), migration (Figure 5C) and VEGF production (Figure 5D). These results imply the involvement of Akt-mTOR signaling in gremlin's actions.

Similarly, in the primary murine RPE cells, the Akt specific inhibitor perifosine or the mTOR kinase inhibit AZD8055 suppressed gremlin-induced cell proliferation (Figure 5E), migration (Figure 5F) and VEGF production (Figure 5G). Collectively, these results demonstrate that activation of Akt-mTOR signaling, downstream of VEGFR2, is required for gremlin-exerted pleiotropic functions in RPE cells.

### mTORC2 activation mediates gremlin-induced pleiotropic functions in RPE cells

**Figure 6:**
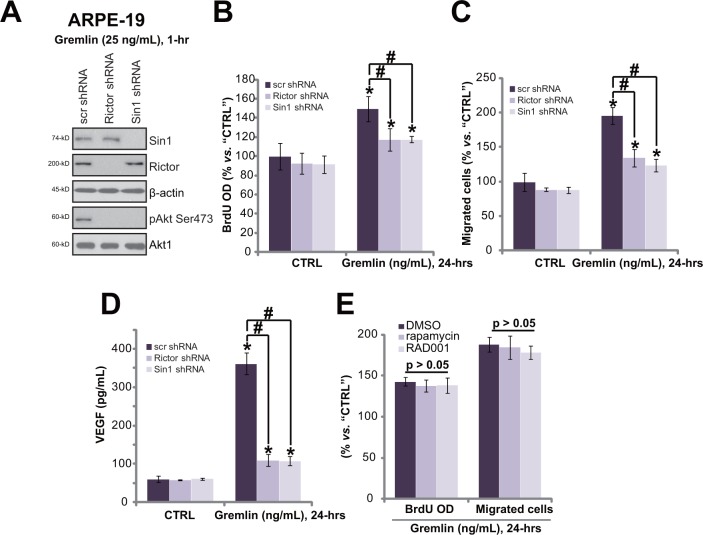
mTORC2 activation mediates gremlin-induced pleiotropic functions in RPE cells Stable APRE-19 cells with the scramble control non-sense shRNA (“scr shRNA”), Rictor shRNA or Sin1 shRNA were treated with gremlin (25 ng/mL) for applied time, expression of listed proteins **A.**, cell proliferation **B.**, migration **C.** and VEGF production **D.** were tested. APRE-19 cells were pre-treated for 30 min with the mTORC1 inhibitor rapamycin (500 nM) or RAD001 (500 nM), cells were then treated with gremlin (25 ng/mL) for 24 hours, relative BrdU incorporation and cell migration were tested **E.**. “DMSO” stands for 0.1% DMSO vehicle control. Experiments in this figure were repeated three times, and similar results were obtained. * *p* < 0.05 *vs.* “CTRL” group. ^#^ *p* < 0.05.

AZD8055 [18] and OSI-027 [19] are both mTOR kinase inhibitor, which block mTORC1 and mTORC2 simantanuously [20]. We analyzed the role of each single mTOR complex in gremlin's activities. To study the involvement of mTORC2, shRNA method was applied to selectively and stably knockdown its component Rictor or Sin1 [20, 21]. Western blot results in Figure 6A confirmed Rictor or Sin1 knockdown by targeted-shRNA in APRE-19 cells. As a result, p-Akt Ser473, the indicator of mTORC2 activation [22, 23], was largely inhibited (Figure 6A). Remarkably, Rictor or Sin1 knockdown significantly inhibited gremlin-induced cell proliferation (Figure 6B), migration (Figure 6C) and VEGF production (Figure 6D) in APRE-19 cells. On the other hand, two known mTORC1 inhibitors, rapamycin and RAD001 [24], showed no significant effect on gremlin-induced cell proliferation (Figure 6E) and migration (Figure 6E). These results imply that activation of mTORC2 is required for gremlin-exerted pleiotropic functions in ARPE-19 cells.

## DISCUSSION

ROP is characterized by late-phase pathologic vasoproliferation [3]. Existing evidences have proposed gremlin as a novel VEGFR2 agonist, which promotes angiogenic responses [6, 13]. In the present study, we discovered that gremlin expression was significantly increased in the retinas of ROP model mice. Intriguingly, its level was correlated with VEGF upregulation. Further *in vitro* studies in primary and established RPE cells showed that gremlin activated VEGFR2-Akt-mTORC2 signaling, and promoted cell proliferation, migration and VEGF production. Pharmacological and/or genetic blockage of VEGFR2-Akt-mTORC2 largely attenuated gremlin-mediated pleiotropic functions in RPE cells. Thus, VEGFR2-Akt-mTORC2 activation mediates gremlin's activities in RPE cells.

mTOR lies in two multi-protein complexes, including rapamycin-sensitive mTORC1 and later-discovered mTORC2 [23, 25, 26]. mTOR1, containing mTOR, Raptor, PRAS40 and possible several others, phosphorylates S6K1 and 4E-BP1, and its activity could be inhibited by rapamycin or its analogs (*i.e.* RAD001) [23, 25, 26]. Rapamycin-insensitive mTORC2, on the other hand, is composed of mTOR, Rictor, Sin1 and Protor [23, 25, 26]. Existing evidences have demonstrated that both complexes, depending on stimuli and/or cell types, could be important for cell proliferation, migration and angiogenesis [23, 25, 26].

In the current study, we showed that mTORC2 likely plays a more important role in mediating gremlin-mediated pleiotropic functions in RPE cells. Inhibition of this complex, via shRNA knockdown of mTORC2 component rictor or Sin1, largely attenuated gremlin-induced RPE cell proliferation, migration and VEGF production. Interestingly, the well-established mTORC1 inhibitors (rapamycin and RAD001) showed almost no effect on gremlin's activities in RPE cells. These results are in line with recent studies showing an important role of mTORC2 in promoting cell proliferation, migration and angiogenesis [22, 27]. The detailed mechanisms warrant further characterizations.

Studies have confirmed the pivotal role of VEGF in the development of ROP [3, 28]. Anti-VEGF therapies have displayed some benefits in the treatment of ROP [3, 28]. Our *in vivo* results showing that gremlin level was increased in ROP model mice retinas, which was correlated with VEGF upregulation. The *in vitro* studies showed that gremlin could promote RPE cell proliferation, migration and more importantly, VEGF production. These results imply that anti-gremlin therapy may provide an alternative treatment for ROP along with other proliferative retinopathies.

## MATERIALS AND METHODS

### Reagents and chemicals

Gremlin, RAD001, rapamycin and perifosine were purchased from Sigma Chemicals (Shanghai, China). AZD8055 and OSI-027 were purchased from Selleck (Nanjing, China). All phosphorylation antibodies and their non-phosphorylated controls were obtained from Cell Signaling Tech (Danvers, MA). Anti-VEGF antibody and all other antibodies were purchased from Santa Cruz Biotech (Santa Cruz, CA).

### ARPE-19 cell culture

As described [15], human RPE ARPE-19 cells were maintained in DMEM/Nutrient Mixture F-12 (DMEM/F12, Gibco Life Technologies, Shanghai, China) with 10% fetal bovine serum (FBS) (Hyclone), penicillin/streptomycin (1:100, Sigma, St. Louis, MO), and 4 mM L-glutamine and 0.19% HEPES, in a humidified incubator at 37°C and 5% CO_2_.

### Primary murine RPE cell isolation and culture

The primary culture of murine RPE cells was performed in accordance with the Institutional Animal Care and Use Committee (IACUC). The protocols were approved by the Ethics Committee and Internal Review Board (IRB) of all authors institutions. All mice utilized in this study were purchased Soochow University Animal Facility (Suzhou, China). Three-to-four week old C57/B6 mice were anesthetized, and the eyeballs were taken out and diluted in D-hank's buffer (Sigma). The retinas were striped out carefully. Parenzyme (0.125%) was then added to digest for 30 min, before adding the complete medium (80% DMEM/F-12, 20% FBS) to terminate digestion. Afterwards, the supernatants were centrifuged twice and primary murine RPE cells were cultured in the complete medium.

### Methylthiazol tetrazolium (MTT) assay

The routine MTT assay [15] was performed to test cell proliferation after applied treatments. MTT optic density (OD) at 490 nm was recorded as a quantitative indicator of cell proliferation.

### BrdU incorporation assay

The RPE cell proliferation was also tested via the incorporation of 5-bromo-2'-deoxyuridine (BrdU). Briefly, BrdU (10 μM) was initially added to the medium, and then the cells were subjected to applied treatment/s. Afterwards, BrdU incorporation was determined via BrdU ELISA kit (Roche Diagnostics, Shanghai, China) according to the protocol provided. The BrdU ELISA OD was tested as a quantitative measurement of cell proliferation.

### “Transwell” assay for cell migration

As described in our previous study [15], the “Transwell” assay was performed to test cell migration in the modified Corning chambers (Corning, Lowell, MA). RPE cells were allowed to migrate for 24 hours. The number of migrant RPE cells attaching to the lower surface was counted under microscopy. Mitomycin C (10 μg/mL, Sigma) was added to prevent cell proliferation [15].

### Real-time quantitative PCR (“RT-qPCR”) assay

RNA was extracted via the Trizol (Invitrogen) method, and reverse transcription was performed through the TOYOBO ReverTra Ace-a RT-PCR kit (TOYOBO, Japan). The ABI7700 system (Applied Biosystems) was utilized for real-time quantitative PCR assay. Glyceraldehyde-3-phosphate dehydrogenase (GAPDH) primers were forward, 5′-GAAGGTGAAGGTCGGAGTC-3′; reverse, 5′-GAAGATGGTGATGGGATTTC-3′; The primers for VEGF were described [29, 30]. The primers for gremlin were also described [31]. The melt curve analysis was performed for analyzing melting temperature. Relative gremlin or VEGF mRNA expression was calculated via the comparative Ct method (2^−ΔΔCt^) [32], with GAPDH as the reference gene.

### VEGF ELISA assay

After treatment of cells, the conditional medium was collected. A commercial VEGF ELISA kit (R&D Systems, Nanjing, China) utilizing a sandwich two-site immunoassay was applied to test the VEGF content in the medium.

### Western blots analysis

The detailed protocol for the Western blot assay was previously described [15]. The total gray of each protein band was quantified via the ImageJ software, and the value was normalized to each loading control.

### VEGFR2 shRNA knockdown

VEGFR2 shRNAs were designed, synthesized and verified by Genepharm (Shanghai, China). The lentiviral shRNA was produced by constructing a GV248 vector (Genechem, Shanghai, China) with a puromycin resistance gene and either scramble control shRNA, or shRNA to VEGFR2 (two shRNAs, VEGFR2 shRNA-1 or VEGFR2 shRNA-2). The lentivirus (10 μL/mL medium) was added directly to cultured RPE cells. Stable cells were selected by puromycin (5 μg/mL, Sigma) for 10-12 days until single resistant colony was formed. VEGFR2 knockdown in stably cells was verified by Western blot assay.

### Rictor or Sin1 shRNA knockdown

The lentiviral particles with Rictor shRNA (sc-61478-V), Sin1 shRNA (sc-60984-V) and scramble non-sense control shRNA (sc-108080) were purchased from Santa Cruz Biotech (Nanjing, China). The particles (10 μL/mL medium) was added to the cultured cells. Stable cells were again selected by puromycin. The knockdown of targeted protein (Rictor or Sin1) was again tested by Western blot assay.

### Mice retinopathy of prematurity (ROP) model

The weight-matched C57BL/6J mice (P7) were utilized. To achieve ROP, mice were maintained in 75% oxygen chamber (Oxycycler; Biospherix, Lacona, NY) for 5 days (P7-P12). Afterwards, mice were kept in normoxia for additional 5 days. Control mice were maintained in normoxia. At P17, mice were anesthetized, and the eyeballs were taken out. The retinas were striped out carefully, which were incubated in lysis buffer as described. mRNA and protein expressions of gremlin and VEGF were tested by RT-qPCR assay and Western blot assay respectively. The protocols were again in accordance with the IACUC, and were approved by the Ethics Committee and IRB of all authors institutions.

### Statistics

Statistically significant differences were determined by one-way ANOVA and were defined as *p* <0.05. All experiments were repeated independently at least three times.
